# Genetic Variation in the von Willebrand Factor Gene in Swedish von Willebrand Disease Patients

**DOI:** 10.1055/s-0037-1618571

**Published:** 2018-01-30

**Authors:** Eric Manderstedt, Christina Lind-Halldén, Stefan Lethagen, Christer Halldén

**Affiliations:** 1 Department of Environmental Science and Bioscience, Kristianstad University, Kristianstad, Sweden; 2 National Haemophilia Center, University Hospital, Rigshospitalet, Copenhagen, Denmark; 3 Department for Coagulation Disorders, University Hospital, Malmö, Sweden; 4 Sobi, Stockholm, Sweden

**Keywords:** DNA, molecular, diagnosis, Sweden, von Willebrand disease, von Willebrand factor

## Abstract

von Willebrand factor (VWF) level and function are influenced by genetic variation in *VWF* and several other genes in von Willebrand disease type 1 (VWD1) patients. This study comprehensively screened for *VWF* variants and investigated the presence of *ABO* genotypes and common and rare *VWF* variants in Swedish VWD1 patients. The *VWF* gene was resequenced using Ion Torrent and Sanger sequencing in 126 index cases historically diagnosed with VWD. Exon 7 of the *ABO* gene was resequenced using Sanger sequencing. Multiplex ligation-dependent probe amplification analysis was used to investigate for copy number variants. Genotyping of 98 single nucleotide variants allowed allele frequency comparisons with public databases. Seven VWD2 mutations and 36 candidate VWD1 mutations (5 deletions, 4 nonsense, 21 missense, 1 splice, and 5 synonymous mutations) were identified. Nine mutations were found in more than one family and nine VWD1 index cases carried more than one candidate mutation. The T-allele of rs1063857 (c.2385T > C, p.Y795 = ) and blood group O were both frequent findings and contributed to disease in the Swedish VWD1 population. VWD2 mutations were found in 20 and candidate VWD1 mutations in 51 index cases out of 106 (48%). *VWF* mutations, a *VWF* haplotype, and blood group O all contributed to explain disease in Swedish VWD1 patients.

## Introduction

von Willebrand disease (VWD) is often caused by genetic defects in the von Willebrand factor ( *VWF* ) gene and is divided into three major subtypes: type 3 (VWD3) is characterized by total absence of VWF, type 2 (VWD2) by functional disturbance of VWF, and type 1 (VWD1) by low plasma concentration of functionally normal VWF. 1 Diagnosis of VWD is challenging due to the heterogeneity of the disease and can be made based on phenotypic testing alone; therefore, mutation analysis has not been regarded as necessary. 2

The highly polymorphic *VWF* gene spans 178 kb, is composed of 52 exons that vary in size from 50 bp to 1.3 kb, and gives rise to an mRNA that is ∼9 kb in size. There is an unprocessed pseudogene ( *VWFP1* ) located on chromosome 22 with 97% sequence homology to the *VWF* gene sequence for exons 23 to 34. The large size, the highly polymorphic sequence, and the presence of the pseudogene complicate screening for pathogenic variants in *VWF* . *VWF* mutations have been reviewed, 1 and they are compiled in the von Willebrand factor Variant Database ( http://www.vwf.group.shef.ac.uk/ ) containing >700 unique variants. VWD1 mutations are mostly missense mutations that are spread throughout the whole *VWF* gene and many show incomplete penetrance. Previous mutation screening in VWD1 identified *VWF* mutations in 105 of 150 (70%) patients, but 57 of these patients had abnormal multimers and VWD2, leaving 93 VWD1 patients with qualitatively normal multimers. 3 In these patients fewer mutations were identified (51 of 93, 55%). In a similar study, 150 Canadian VWD patients were analyzed for mutations in the *VWF* gene. 4 The authors identified putative mutations within the *VWF* gene in 78 patients (63%) with qualitatively normal multimers. In both studies, the contributions of other factors such as ABO blood group were more important in milder cases and patients with lower VWF levels had a higher probability of carrying mutations in the *VWF* gene itself. The mutations act through two major mechanisms: intracellular retention 5 and rapid clearance; 6 however, the molecular mechanisms of a substantial number of candidate mutations are still unknown, creating an uncertainty in the classification of VWD1 mutations. Investigating the genetic variation of the *VWF* gene in healthy controls of African American descent identified several missense variants that were associated with VWF level variation, but also showed high frequencies of certain rare missense variants previously reported as VWD1 mutations that were not associated with VWF levels. 7 8 9 This emphasizes the challenge in determining the pathogenicity of candidate *VWF* mutations.

Meta-analysis of several genome-wide association studies 10 identified single nucleotide variants (SNVs) in the *VWF* locus that were significantly associated with VWF level variation. The most significant of these was rs1063857 ( *p* = 1.7 × 10 ^−32^ , c.2385T > C, p.Y795 = ) located in a ∼30 kb region defined by 47 significant SNVs. It also identified genes other than *VWF* that contribute to plasma level variation of VWF in the normal population. 10 Comparing the effects of these different genetic factors on level of VWF, the *ABO* blood group was the largest contributor explaining ∼25% of VWF level variation with much smaller contributions from the other factors including the *VWF* gene. 10 According to Johnsen et al, 8 rare *VWF* missense variants in aggregate explained 3.3% of the VWF phenotypic variance, whereas the common *VWF* variants reported by Campos et al 11 explained 0.9 to 1.5%. Since the previously identified common variants explained only a small fraction of the VWF level variation, several normal populations were reanalyzed with Human Exome Bead Chips aimed to investigate rarer coding variants. 12 VWF levels were affected by a single rare variant in *STAB2* and by multiple rare or low-frequency variants within *ABO* and surrounding genes.

This study aims to identify candidate *VWF* mutations in a population of Swedish patients clinically diagnosed with VWD and simultaneously define VWD1 patients with and without *VWF* mutations. In addition, blood group O status and the potential influence of common *VWF* variants on VWD1 status will be investigated.

## Materials and Methods

### Study Populations and von Willebrand Disease Phenotyping

The VWD study population was recruited at the Department for Coagulation Disorders, Malmö University Hospital (Malmö, Sweden). The population consisted of consecutive patients and their relatives who attended the clinic between the years 1988 and 2005 and corresponds to ∼1,000 individuals belonging to 126 families. This population represents the majority of all families diagnosed with VWD in Sweden during this time period. Approximately 50% of all families were represented by a single index case; the remaining families were represented by 2 to 15 family members. Complete mutation analysis was initially performed on one index case from each of the 126 families. In a second step, the analysis was extended to a total of 288 individuals recruited unevenly from the families. The coagulation department in Malmö is a national referral center for bleeding disorders, and all patients' details are collected in a register. Clinical and laboratory data were recorded for each patient and their bleeding phenotypes were classified. 13 We used historical VWF levels usually determined at the time of the original diagnostic work-up. Therefore, different phenotypical methods have been used. VWF activity was measured using the traditional VWF: ristocetin cofactor (VWF:RCo) method based on aggregation of platelets or an automated VWF:RCo assay based on the BCS coagulation analyzer using the BC von Willebrand reagent (Dade Behring Inc., Newark, Delaware, United States). VWF antigen (VWF:Ag) levels were measured with electroimmunoassay (the Laurell's method) and immunoradiometric assay, enzyme-linked immunosorbent assay, or line immunoassay. Multimer analysis and ristocetin-induced platelet aggregation were only performed occasionally. There were no further analyses of VWF levels in this study and the historical VWF levels available do not allow further analyses of VWF levels. In a strict sense, not all index cases fulfilled the diagnostic criteria according to ISTH, 14 but at the time of diagnosis, their bleeding symptoms in combination with lowered VWF levels were interpreted as being VWD1. A total of 29 of these families have previously been analyzed for cosegregation between polymorphisms in the *VWF* gene and VWD. 13 The analyses of the populations were approved by the Ethics Committee of the Medical Faculty, Lund University, and the Swedish Data Inspection Board. Written informed consent was obtained from all subjects.

### Ion Torrent Sequencing

Genomic DNA was isolated from whole blood using QIAamp DNA Mini or Maxi Kits (Qiagen GmbH, Hilden, Germany). The DNA concentrations were determined by fluorometry using PicoGreen (Molecular Probes; Invitrogen, Eugene, Oregon, United States) and all samples were normalized to the same concentration. The Ion Torrent sequencing system (Life Technologies, Carlsbad, California, United States) was used for mutation screening. The primer sets were designed using Ion AmpliSeq Designer ( http://www.ampliseq.com , pipeline version 2.2.1). A total of 79 primer pairs that generated amplicons covering 1 kb promoter and 98% of the coding sequence of *VWF* were obtained ( Supplementary Table S1 ). The homologous regions of *VWF* and its pseudogene *VWFP1* were compared using Ensembl BLAST ( http://www.ensembl.org/Multi/blastview ) to ascertain that the primers could differentiate between the two genes. The DNA samples were individually barcoded using Ion Xpress Barcode Adapters. Initial amplification of the targeted regions was performed using the Ion AmpliSeq Library Kit 2.0. Emulsion polymerase chain reaction (PCR) was performed on the OneTouch 2 system with the Ion PGM Template Hi-Q View OT2 kit. Sequencing was performed on an Ion Torrent PGM using Ion PGM Hi-Q View Sequencing kit and Ion PGM 316 chip v2 according to the manufacturer's instructions (Life Technologies). The sequences were aligned against the human reference sequence using Torrent Suite 5.0.5 and variant calling performed using variant calling parameters tuned for AmpliSeq panels. Annotation of the SNVs was accomplished by submitting them to Ensembl Variant Effect Predictor ( http://grch37.ensembl.org/Homo_sapiens/Tools/VEP ), Condel 2.0 ( http://bg.upf.edu/fannsdb/ ), MutationTaster ( http://www.mutationtaster.org/ ), Human Splicing Finder ( http://www.umd.be/HSF3/ ), and NNSPLICE V0.9 ( http://www.fruitfly.org/seq_tools/splice.html ).

### Sanger Sequencing

Sanger sequencing was used to resequence the complete exons 26 and 28 and confirm all putative mutations discovered with Ion Torrent sequencing. The human genomic sequence was obtained from genomic build GRCh37p13. Primers for the 52 different exons of the *VWF* gene and exon 7 of the *ABO* gene ( Supplementary Table S2 ) were designed using NCBI Primer-BLAST ( http://www.ncbi.nlm.nih.gov/tools/primer-blast/index.cgi ) and ordered from DNA Technology A/S (Risskov, Denmark). The samples were sequenced using Big Dye Terminator chemistry, v3.1 (Applied Biosystems, Foster City, California, United States) on an ABI 3130xl sequencer. PCR was performed using KAPA Taq HotStart DNA PCR kit (KAPA Biosystems, Cape Town, South Africa). The primary PCR products were purified with ExoSAP-IT PCR Product Cleanup (Affymetrix, Santa Clara, California, United States), unincorporated dye terminators removed by BigDye XTerminator Purification Kit and sequence data assembled and compared using SeqScape (Applied Biosystems).

### Single Nucleotide Variant Genotyping

Genotypes were determined for 98 common SNV markers (95 with minor allele frequencies [MAFs] > 5%) using the Sequenom MassARRAY MALDI-TOF system (Sequenom Inc., San Diego, California, United States) ( Supplementary Table S3 ). Assay design was made using the SpectroDESIGNER software (Sequenom Inc.). Primers were obtained from Metabion GmbH (Martinsried, Germany). The subsequent steps including the Sequenom MassEXTEND reaction were then performed as described by the manufacturer ( http://www.sequenom.com/ ).

### Multiplex Ligation-Dependent Probe Amplification Analysis

Multiplex ligation-dependent probe amplification (MLPA) was used to search for deletions and duplications affecting the complete gene or specific exons. Two MLPA kits that together covered all exons of the *VWF* gene (P011-VWF, P012-VWF; MRC-Holland, Amsterdam, The Netherlands) were used according to the manufacturer's instructions ( http://www.mrc-holland.com ). The MLPA analysis used 50 ng of template DNA and the PCR products were separated on an ABI 3130xl automatic sequencer and analyzed using Coffalyser (MRC-Holland).

### Genetical and Statistical Analyses

Variant data for genomic regions was taken from the CEU population of the 1000 Genomes project ( http://ftp.1000genomes.ebi.ac.uk/vol1/ftp/release/20110521/ ). The CEU population consists of 99 Utah residents with northern and western European ancestry, and has been analyzed extensively in both the HapMap and 1000 Genomes projects. For exonic variants, data were compared with data from the European part of the Exome Aggregation Consortium (ExAC; http://exac.broadinstitute.org ), which contains data from ∼33,000 European individuals. All variants identified in the VWD population were compared with the information in these databases and in the von Willebrand factor Variant Database ( http://www.vwf.group.shef.ac.uk/ ) listing all known *VWF* mutations. Statistical analyses were made using R statistical software. 15 All SNVs were evaluated for Hardy–Weinberg's equilibrium and then allele frequencies were tested for association with VWD1 using homogeneity tests based on the asymptotic chi-square distribution. Haploview 16 was used to characterize the linkage disequilibrium pattern in the *VWF* gene region using data from the CEU population of the 1000 Genomes database. This defined haploblocks, their cosegregation rates, and the underlying haplotypes in each region. Haplotypes of the VWD1 population were constructed with PHASE v2.1. 17

## Results

All coding exons of the *VWF* gene were screened for variants using an AmpliSeq strategy and the Ion Torrent platform. This sequencing was performed for all exons except exon 26 on a total of 126 index cases from presumably unrelated Swedish families. Sanger sequencing was used to resequence the complete exons 26 and 28 in all index cases and confirm all mutation candidates. The Ion Torrent sequencing data covered 100% of the exonic positions except those of exon 26. The read depth was > 100 *X* in > 95% of individuals for > 98% of positions. The Sanger sequence data were generally of high quality with > 95% of bases having a Phred score of 30 or higher in > 95% of individuals.

**Fig. 1:**
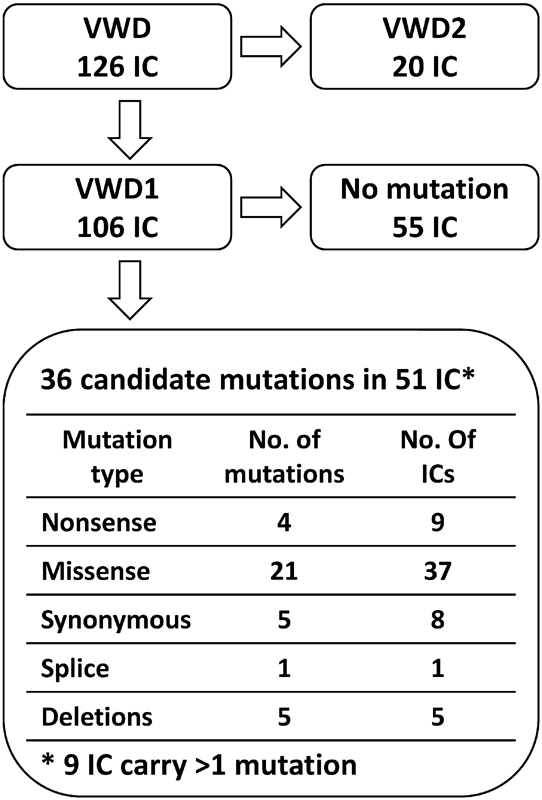
The mutation status of the 126 von Willebrand disease (VWD) index cases (IC).

Seven variants are listed explicitly as VWD2 mutations in the von Willebrand factor Variant Database ( http://www.vwf.group.shef.ac.uk/ ). These were found in 20 families ( Table 1 , Fig. 1 ). One VWD2 mutation was particularly common and was present in more than 50% of the families (11 out of 20 families). It is interesting to note that one of the VWD2 mutations (p.G1672R) occurred together with a known VWD1 mutation (p.P1413L) in three out of three families, indicating that these families most likely have a common origin and that these two mutations occur on the same haplotype. In the remaining 106 index cases, a total of three large and two small deletions and 39 SNVs with a MAF < 0.5% were identified in the coding sequence ( Table 2 ). There were four nonsense variants identified in a total of 9 index cases and 25 missense variants identified in a total of 41 index cases. In addition, there were nine synonymous variants in a total of 15 index cases and one variant potentially affecting splicing in 1 index case. A total of three large and two small deletions were detected by Ion Torrent sequencing. MLPA analysis confirmed the three large deletions; one encompassing exons 14 to 52 and two with partial deletions of exon 28. All were present in one index case each.

**Table 1:** Type 2 VWD mutations identified among the 126 index cases

SNP ID	Chr. position a	Base change	Exon	Amino acid change	Index cases	MAF in ExAC b	In silico prediction c	VWD type in database d
rs61748466	6155892	c.2278C > T	17	p.R760C	1	0.00002	3	2N
rs267607326	6132007	c.3437A > G	26	p.Y1146C	1	0	4	2A
rs61748511	6131999	c.3445T > C	26	p.C1149R	1	0	4	2A
rs61749395	6128641	c.3943C > T	28	p.R1315C	2	0.00002	4	2A/M
rs61750071	6128464	c.4120C > T	28	p.R1374C	11	0.00002	4	2A/M
rs61750100	6128067	c.4517C > T	28	p.S1506L	1	0	4	2A
rs61750598	6127570	c.5014G > A	28	p.G1672R	3	0.0007	1	2A

Abbreviations: Chr., chromosome; ExAC, Exome Aggregation Consortium; MAF, minor allele frequency; SNP, single nucleotide polymorphism; VWD, von Willebrand disease.

a According to GRCh37p13.

b MAF observed in 33,000 individuals from the non-Finnish European population in the ExAC database.

c For missense mutations, the predictions are classified according to damaging (1) or tolerated (0) using the programs SIFT, PolyPhen-2, Condel 2.0, and MutationTaster. The table presents the sum of all predictions.

d von Willebrand factor Variant Database, previously ISTH-SSC VWF Online Database.

**Table 2:** Type 1 von Willebrand disease mutations/neutral variants identified among the 126 index cases

SNP ID	Chr. position a	Base change	Exon	Amino acid change	Index cases	MAF in ExAC b	In silico prediction c	VWD type in database d
Nonsense								
rs61750595	6127609	c.4975C > T	28	p.R1659*	4	0.00008	–	3
New	6094247	c.6940C > T	40	p.Q2314*	1	0	–	
rs61751296	6078503	c.7603C > T	45	p.R2535*	3	0.00005	–	3
New	6058958	c.8247C > A	51	p.Y2749*	1	0	–	
Missense								
New	6204703	c.580A > G	6	p.S194G	1	0	1	
rs199570108	6181528	c.1078G > A	9	p.G360S	1	0.0002	4	
rs41276738	6143978	c.2561G > A	20	p.R854Q	2	0.004	3	1
rs267607328	6131977	c.3467C > T	26	p.T1156M	1	0	4	1
rs61749367	6128898	c.3686T > G	28	p.V1229G	2	0.001	0	1
rs61749368	6128892	c.3692A > C	28	p.N1231T	2	0.001	1	1
rs61750074	6128449	c.4135C > T	28	p.R1379C	1	0.00002	3	1
rs61750079	6128346	c.4238C > T	28	p.P1413L	1	0.00001	4	1
rs1800386	6127833	c.4751A > G	28	p.Y1584C	11	0.004	3	1
rs61750604	6125715	c.5278G > A	30	p.V1760I	1	0.001	0	1
New	6122758	c.5509C > T	32	p.R1837W	1	0	4	
New	6121281	c.5636G > T	33	p.C1879F	1	0	3	
New	6121256	c.5661G > T	33	p.K1887N	1	0	1	
New	6120827	c.5798C > T	34	p.P1933L	1	0	3	
rs144072210	6105380	c.5851A > G	35	p.T1951A	1	0.001	1	
New	6103210	c.6416G > A	37	p.C2139Y	1	0	4	
New	6094737	c.6893C > T	39	p.T2298M	1	0.0002	3	
rs764980510	6092417	c.6980G > A	41	p.C2327Y	4	0.00003	4	
rs144769404	6092390	c.7007C > T	41	p.P2336L	1	0.0002	0	
New	6090995	c.7244C > T	42	p.T2415I	1	0	3	
New	6085371	c.7343G > A	43	p.C2448Y	1	0	4	
rs61751286	6085324	c.7390C > T	43	p.R2464C	1	0.00006	3	1
rs61751289	6085284	c.7430G > A	43	p.C2477Y	1	0	4	1
New	6078427	c.7679G > T	45	p.G2560V	1	0	4	
rs61751302	6062708	c.7940C > T	48	p.T2647M	1	0.003	1	1
Synonymous and splice								
rs2229444	6219682	c.390C > T	5	SYN	3	0.003	1	
rs143054357	6204737	c.546G > A	6	SYN	1	0.001	2	
New	6173411	c.1432 + 1G > T	12	Splice	1	0	2	
New	6128504	c.4080C > T	28	SYN	1	0	2	
rs140464171	6128438	c.4146G > T	28	SYN	2	0.0009	2	Polym.
New	6128162	c.4422A > G	28	SYN	1	0	1	
rs144796763	6128141	c.4443G > T	28	SYN	1	0.002	1	
rs55784921	6103738	c.6099C > T	36	SYN	2	0.002	1	Polym.
rs55944252	6085370	c.7344C > T	43	SYN	1	0.0003	2	Polym.
rs41276732	6061593	c.8079C > T	49	SYN	3	0.003	2	
Deletion/duplication								
New		Deletion	14–52	–	1	–	–	
New	6166017	c.1931_1945 e	15	–	1	–	–	
New	6128169	c.4414delG	28	p.D1472Tfs*53	1	0	–	
New		Deletion	28	–	1	–	–	
New		Deletion	28	–	1	–	–	

Abbreviations: Chr., chromosome; ExAC, Exome Aggregation Consortium; MAF, minor allele frequency; SNP, single nucleotide polymorphism; VWD, von Willebrand disease.

a According to GRCh37p13.

b MAF observed in 33,000 individuals from the non-Finnish European population in the ExAC database.

c For missense mutations, the predictions are classified according to damaging (1) or tolerated (0) using the programs SIFT, PolyPhen-2, Condel 2.0, and MutationTaster. Synonymous changes are classified as potential alteration of splicing (1) or not (0) using Human Splicing Finder and NNSPLICE. The table presents the sum of all predictions.

d von Willebrand factor Variant Database, previously ISTH-SSC VWF Online Database.

e Complete base change is c.1931_1945 + 5delAGCCAGGCCGCTGTGGTGCG.

Two of the nonsense variants are annotated as VWD3 mutations in the von Willebrand factor Variant Database and these were present in three (p.R2535*) and four (p.R1659*) index cases, respectively ( Table 2 , Fig. 1 ). The remaining two nonsense variants were not previously described and were found in one index case each. Eleven out of the 25 missense variants are annotated as VWD1 mutations in the von Willebrand factor Variant Database. Out of the remaining 14 missense variants, 10 were classified as damaging by at least three out of four prediction programs (SIFT, PolyPhen-2, Condel 2.0, and MutationTaster). For the nine synonymous variants, the potential for inducing alternative splicing was evaluated with Human Splice Finder and NNSPLICE. Five were defined as affecting splicing by both programs, among those were c.4146G > T that have previously been reported by Mufti et al 18 as a splicing variant. The p.Y1584C mutation was present in 11 index cases and was thus the most common VWD1 mutation in the Swedish population. The nonsense and missense mutations that were detected in the Swedish population are primarily located in the latter part of the *VWF* gene, from exon 28 onward ( Table 2 ). Thirteen index cases carried more than one variant, 11 index cases had two variants, and 2 index cases had three variants ( Table 3 ). Eight out of these had at least one mutation described in the von Willebrand factor Variant Database (marked in bold in Table 3 ).

**Table 3:** Index cases with two or three candidate mutations/variants

Index case	Candidate mutation 1		Candidate mutation 2	
	Base change	Amino acid change	Base change	Amino acid change
59	c.8247C > A	p.Y2749*	**c.4751A** **>** **G**	**p.Y1584C**
46	**c.7603C** **>** **T**	p.R2535*	c.8079C > T	SYN
87	c.6980G > A	p.C2327Y	c.7679G > T	p.G2560V
57 a	c.6940C > T	p.Q2314*	c.580A > G	p.S194G
33	c.6893C > T	p.T2298M	**c.6099C** **>** **T**	**SYN**
16	c.5851A > G	p.T1951A	c.546G > A	SYN
64	c.5636G > T	p.C1879F	c.8079C > T	SYN
56	c.5509C > T	p.R1837W	c.390C > T	SYN
36 b	**c.4975C** **>** **T**	p.R1659*	**c.2561G** **>** **A**	**p.R854Q**
93	**c.4751A** **>** **G**	**p.Y1584C**	c.7244C > T	p.T2415I
38	**c.4751A** **>** **G**	**p.Y1584C**	c.390C > T	SYN
73	**c.3686T** **>** **G**	**p.V1229G**	**c.3692A** **>** **C**	**p.N1231T**
5	**c.3686T** **>** **G**	**p.V1229G**	**c.3692A** **>** **C**	**p.N1231T**

a Also carries the variant c.390C > T (SYN).

b Also carries the variant c.4146G > T (SYN).

After identification of index cases with (51) and without (55) candidate mutations as defined earlier ( Fig. 1 ), the historical phenotypic data were used to investigate VWF levels and activities among these two groups of patients. VWF levels are given as IU/dL and the patients with mutations showed in general lower values (VWF:Ag; median 34, minimum 10, and maximum 63) than patients without identified VWF mutations (VWF:Ag; median 42, minimum 27, and maximum 59). The corresponding VWF activity values were (VWF:RCo; median 37, minimum <4, and maximum 71) for patients with mutations and (VWF:RCo; median 48, minimum 29, and maximum 56) for patients without mutations.

The mutation analysis was extended to include a total of 288 individuals from the 126 families. This was made to allow cosegregation analysis of the candidate mutations with disease in families where this was possible. Complete cosegregation was observed for all type 2 VWD families that could be evaluated. In contrast, incomplete cosegregation was observed for 4 out of 17 investigated type 1 VWD families. However, this analysis is difficult to interpret due to the low and varying number of individuals analyzed in the different families.

Association analysis comparing the 106 VWD1 patients with the CEU population of the 1000 Genomes database using 98 common *VWF* SNVs (95 with MAF > 5%) was made to search for SNVs associated with VWD1 status. Only one SNV showed a nominally significant allele frequency difference (rs6489695, *p* = 0.03). This SNV was not significant after Bonferroni's correction and was located ∼100 kb from rs1063857(c.2385T > C, p.Y795 = ) that was identified as the most significant SNV in previous studies. 10 11 The MAF for rs1063857 among VWD1 patients was 30%, whereas it was 35% in the CEU population ( Supplementary Table S3 ). The major T-allele previously associated with lower VWF levels is thus also enriched in the Swedish VWD1 population. 10 11

Blood group O is known to be overrepresented among VWD1 cases, and in this study, 75 index cases (71% of 106 VWD1 index cases) were of type O, clearly higher than the Swedish national average of ∼38% type O. Comparing index cases with or without a putative *VWF* mutation, the type O frequency is slightly higher among the mutation-negative cases (40/55 ≥ 73 vs. 35/51 ≥ 67%) but since the total number of cases is rather low, the observed difference is not significant ( Table 4 ). As expected, a higher VWF:Ag level was correlated with the absence of a candidate mutation and with blood group O. Comparable with other studies, no overrepresentation of type O was found in index cases carrying a VWD2 mutation (25%).

**Table 4:** VWF:Ag levels among Type 1 VWD patients

Mutation and blood group status	VWF:Ag < 30 IU/dL	VWF:Ag 30–50 IU/dL	VWF:Ag > 50 IU/dL
Mutation	21	29	1
No mutation	4	43	8
Blood group O	14	54	7
Blood group non-O	11	18	2

Abbreviations: VWD, von Willebrand disease; VWF:Ag, von Willebrand factor antigen.

**Fig. 2:**
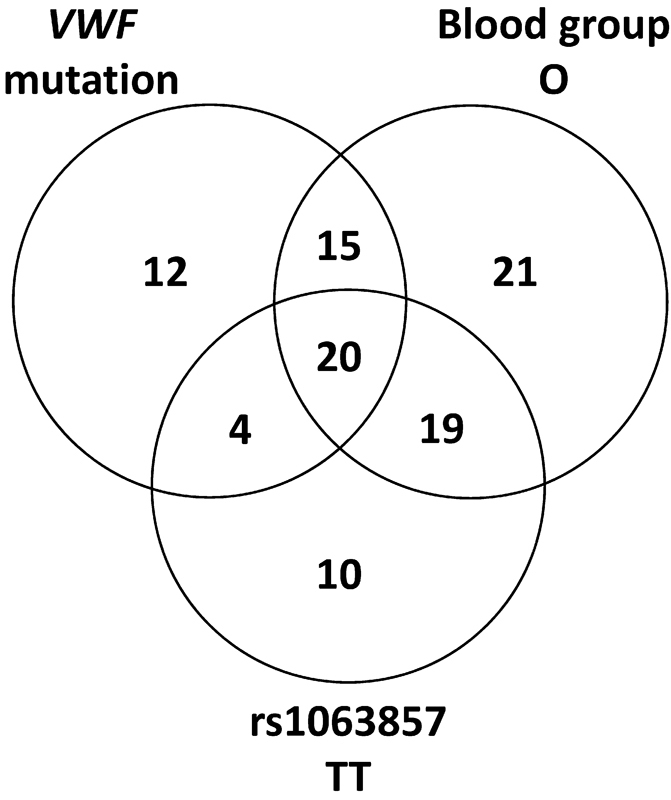
The presence of von Willebrand factor (VWF) mutations, blood group O, and the TT genotype of rs1063857 in the 106 von Willebrand disease type 1 index cases.

The VWD bleeding phenotype is influenced by multiple factors; rare *VWF* mutations with small or large phenotypic effects, common *VWF* variants with small effects, blood group O, and several other genes. Combining the information from our *VWF* mutation analysis with genotyping data of rs1063857 in the *VWF* gene and blood group O, data show that 58 out of 106 index cases in the Swedish VWD1 population have two or more of these factors that have been associated with low VWF levels ( Fig. 2 ). The different factors seem to be distributed randomly among the index cases. This observed independence between factors is also true for the number of index cases lacking any of the factors 5 compared with those that have at least one (101).

## Discussion

Several large association studies identified a 30 to 50 kb region in the *VWF* gene where several common SNVs were significantly associated with VWF level variation. 10 11 The most significant SNV in the study by Smith et al 10 was rs1063857 ( *p* = 1.7 × 10 ^−32^ , c.2385T > C, p.Y795 = ). The effect of this SNV was small, with a one-unit increase in minor allele dosage corresponding to an average increase in the VWF level of 6%. 10 The previously disease-associated T-allele was enriched also in the Swedish population compared with the CEU population (70 and 65%, respectively).

In addition to common variants, there are rare variants that can affect the VWF levels. The contributions of such rare variants can vary in size from the strong effects of *bona fide* mutations that are fully penetrant and capable of explaining disease on their own, to variants with low impact on phenotype that have low penetrance and must work together with other factors to cause disease. The variants with low impact on phenotype are obviously much more difficult to identify as mutations as they are often not cosegregating with disease in the families where they occur. They are also often present in the background population at higher frequencies in comparison with the *bona fide* mutations that often appear as private mutations. Thus, neither the presence of candidate mutations in a mutation database nor their absence in a background population is definitive determinants of their eventual effects. More conclusive evidence can in most cases only be achieved through functional studies using cell cultures or various animal models.

This study detected several mutations present in the von Willebrand factor Variant Database: 11 VWD1, 7 VWD2, and 2 VWD3 mutations. The two most common mutations, p.Y1584C (VWD1) and p.R1374C (VWD2), were found in 11 index cases each in our study. The p.Y1584C mutation has been shown to display increased a disintegrin and metalloproteinase with a thrombospondin type 1 motif, member 13 (ADAMTS13) proteolysis, a loss of high-molecular-weight multimers and a lower capacity to support thrombus formation probably as a result of the increased ADAMTS13 cleavage. 19 This mutation has been found at high frequencies in other studies. It was the most common mutation (15% of investigated families) in a Canadian cohort study 4 and in an English study (25% of families). 20 It was also found in 8% of families in a European study. 3 It has an allele frequency of 0.4% in the ExAC database and is thus highly enriched in populations selected for VWD. In this study, it was detected in 11 out of 126 families (8.7%). In eight of these families, 13 out of 14 mutation-carrying individuals had blood group O. In the remaining three families, the five mutation-carrying individuals all had non-O blood group, but one had the missense mutation p.T2415I and one had the nonsense mutation p.Y2749* that likely contributed to explain the disease phenotype in those cases. Thus, the effect on VWD of p.Y1584C was in most cases supported by blood group O as has been previously reported 1 20 and in some cases by additional mutations. The p.R1374C mutation is characterized by low VWF level and decreased interaction between VWF and platelet GPIb. Patients carrying this mutation have historically been categorized as VWD1, VWD2A, and VWD2M. 21 22 It was one of the five most common mutations in a Spanish study 23 and has also been found in France 24 and Turkey. 25 In this study, it was detected in 11 out of 126 families in a total of 31 diseased individuals.

Two nonsense VWD1 mutations occurred in multiple index cases. The p.R1659* mutation was detected in four VWD1 families in heterozygous form in a total of nine patients. All of these patients had blood group O. The p.R1659* mutation is annotated in the von Willebrand factor Variant Database ( http://www.vwf.group.shef.ac.uk/ ) as a VWD3 mutation. In heterozygous form, it has previously been reported in VWD1 26 and in homozygous form, in VWD3 patients 27 in Finland and Sweden. It has been reported as fairly common in Finland where it together with another mutation explains the majority of VWD3 in Finland. 28 It is the most common nonsense mutation in VWF in the ExAC database being present in five individuals. The p.R2535* mutation was detected in three VWD1 families in heterozygous form in a total of five patients and one individual without disease. Three of the patients also had blood group O, whereas the two remaining patients and the individual without disease had non-O blood group. This mutation is also annotated in the von Willebrand factor Variant Database ( http://www.vwf.group.shef.ac.uk/ ) as a VWD3 mutation in Sweden. 26 This mutation is the second most common nonsense mutation in the ExAC database and represents the type of recessive mutation that can be fairly common in the general population. These two mutations give rise to a mild phenotype in heterozygous form and can operate in a dominant manner giving rise to VWD1, often together with additional factors such as blood group O.

Three missense variants occurred in two index cases each; p.R854Q, 3 p.V1229G, and p.N1231T. 4 29 These are all annotated as mutations in the von Willebrand factor Variant Database. p.R854Q is a common mutation that has previously been reported in many different studies. Both p.V1229G and p.N1231T occur together on the same haplotype in two index cases. They are the result of a gene conversion event introducing SNVs rs118022194 and rs141852043 of *VWFP1* into the *VWF* gene. One missense variant p.C2327Y occurred in four index cases and is not present in the database, but was predicted to be damaging by all four prediction programs. An additional seven previously reported VWD1 missense mutations were also detected in the Swedish study population: The p.T1156M mutation has previously been reported from Sweden 30 and Spain. 31 In vitro coexpression with wild-type and p.T1156M carrying *VWF* showed a dominant negative effect on secretion. The p.R1379C mutation has been identified in Swedish patients as part of the European MCMDM-1VWD study 3 and in a separate study. 32 It has also been reported from Spain, 31 33 but no thorough investigation of its functional consequences has been reported. The p.P1413L and p.V1760I mutations have previously been reported in a European 3 and a Canadian study. 4 Cells transfected with the p.R2464C mutation showed a mild reduction in the amount of secreted VWF and characteristic faster running multimeric bands indicating that this mutation is probably causative. Transfections of mutant constructs of p.C2477Y showed intracellular retention and impaired secretion of VWF together with loss of high-molecular-weight multimers. 5 The p.T2647M mutation has previously been found together with p.S2179F. 34 Since p.S2179F cosegregated with disease and has been described as causing increased clearance; it is more likely the causal mutation.

There were 20 variants discovered in the VWD1 population that were not present in the dbSNP: two nonsense, 10 missense, 1 splice, 2 synonymous, and 5 deletions. The four nonsense and five deletions are obvious candidates for being true mutations and 8 out of 10 missense variants were classified as damaging by at least three of four prediction programs (SIFT, PolyPhen-2, Condel 2.0, and MutationTaster). In addition, five out of nine synonymous variants were predicted to affect splicing by both prediction programs (Human Splicing Finder and NNSPLICE). One of these five variants, c.4146G > T, has previously been reported to affect splicing. 18 The 4 nonsense mutations, the 21 missense mutations either annotated as mutations or predicted to be damaging by three or more prediction programs, one splicing mutation, the five synonymous variants predicted to affect splicing by both prediction programs and the three large and two small deletions were present in a total of 51 index cases. Thus, candidate mutations were found in 51 out of 106 index cases (48%). A recent study in the United States investigated 482 patients historically diagnosed with VWD1. 35 When these patients were retested, 172 patients did not meet the current diagnostic criteria for VWD1 or low VWF level (VWF:Ag < 50 IU/dL or VWF:RCo < 53 IU/dL). There was also no difference in bleeding score whether or not, the current criteria were fulfilled. Complete VWF resequencing showed that 45% of the 482 patients with historical VWD1 diagnosis carried a rare variant in the *VWF* gene compared with 62% of the 310 patients fulfilling the modern criteria. Thus, based on the historical VWD1 diagnosis, their observed mutation discovery frequency (45%) is directly comparable and highly similar to the mutation discovery rate observed in this study (48%).

The presence of the pseudogene sequence covering exons 23 to 34 of the *VWF* gene complicated the screening for mutations in this region. To ascertain that all identified variants in this study were variants in the *VWF* gene and not the effect of cross-hybridization to the pseudogene sequence, all primer sequences contained at least one mismatch to the pseudogene sequence and all systems were optimized to amplify only the *VWF* gene sequences. In addition, all amplicon sequences were designed to contain at least one internal position containing a mismatch between the gene and pseudogene sequences. During the sequence interpretation, all sequences were checked for the presence of contaminating pseudogene alleles. The AmpliSeq strategy did not perform well for exon 26 which was instead analyzed using Sanger sequencing. MLPA analysis revealed two exon 28 deletions and one large deletion encompassing exons 14 to 52. The large deletion has been reported in an earlier study 36 that evaluated the inheritance of SNV markers.
