# Supplementary material for: Genetic Variation in the von Willebrand Factor Gene in Swedish von Willebrand Disease Patients
Source: TH Open. 2018 Jan 30;2(1):e39–48. doi: 10.1055/s-0037-1618571 (PMC6524857; doi:10.1055/s-0037-1618571)
Supplement: Supplementary file 1 — Supplementary Table S1 [file 10-1055-s-0037-1618571-s170015-1.pdf]

Table S1 Ion AmpliSeq primers for *VWF* from AmpliSeq Designer, pipeline version 5.63

	Primer sequence	
Exon	Forward	Reverse
52	AGCCTTTATTGTGGGCTCAGAA	CTAAGCCAGGACTTCCCACCAT
51	AAAAATGCTTCCAGTTTATTTCCCTTCT	CCTGTGGCTGGCTTTATTTGGTTA
50	CTGCAAGACTGAACATAATGACTGAC	GGTGGTCAAGCTGCTCACATTTA
49	AGAGATGTGCCTCAGACACTGA	CTTGTTTCATGGTTCTGCAGATTGT
48	GAAAAAGAAGCCAATACTGAACCAAACT	CTGAGAACTGACAAAAGCTGGTTG
47	GCAGTTTGGGTGGGTGATTTTT	AGGAGACAGGGTATGAGAGTGAG
46	CATTTCTGCTTTACAATGACTTGCCT	CGCTGTCACTTGGAGAACGTAC
45	CCCTAAGTTGCTAAAAAGGCAAAGAAT	GAAACCACCTTCCTGAGAGAAGAG
44	TAACACCAACAGCTGGGTGAAA	ATGAAAATGCCCAGACCAGTGA
43	AAAAAGAACCTTTCTTACCCTTCCTAAGA	GGTTTTATTTATTGCCACATTCTCAGCA
42	GCTCACTGTGGAGTTGACACAG	GCACCCTATAGCATAGCTGAATACTTAC
42	CCAACCAAGCCTTGTAGCACTT	CCCAGTGCTGTGATGAGTATGAG
41	CTCCCAACCCAGATTCAGCTAG	GTAATCTCTGTCTCCATCATCATCACTTAC
40	CTTACCCACCTCCTTTCACACA	TGAGGGTGTCAACAGGAACATG
39	GGACTCTAGGTGCCAGTGTTTG	TCCTTTCAGCCTGCTTTTGTTTG
38	GAAGAGGCCAATCACTGGTGAA	GTCTGATGATTAACCATGTTGAATCAGC
37	GCAGATGGCATAGAATGTGGCT	CCAGGGCCACTCAGTTTATCTTTTT
37	ATCTTCCATCTTATTTGATCCTAACTGGAA	ACTGTTTGCTGAATGCCACAAG
36	TATAAGCATCCAAGAGCCTCAGAGT	TGTGATGAATGTGCAGGAACTCT
35	GTCCAGGTGGTTTTCCTGACAT	CCTGTCTGCATGTAGTAGGCAT
34	GCCACAGGTCTCTTCCACTTTAA	GTAGGCCTGTGTTCAATTCTAGGG
34	CAACAGCCAAGCTGAGCATTG	GGCCTTCGTGCCCTAACAG
33	CCCTGTACATGAGACAGGAAGC	GTCCCTATGTCTCCACTGTTAACC
32	GGGTCTCTTGAATACTATTTTTGTTTCTTTG	TGAGGCTGTATTCAGATGCTGGATATAA
31	GTATTGAGCATATTTAATATCAGCCACAACA	CCTTGGAGTTTCTGAATCATTCAGCT
30	AAACTCCAAGGAACACCCAGTT	TTTTGTGGCTCTACTTGATTCAAAATAATC
29	CCCTAAACGGAACGAGAAAATGC	ACATTGCCCTTGTACTCACGAAG
28	GCAGGAAGACCAGGTCCAGTA	CTTCCTGAACCATTTTAATTTCTTCGCTC
28	GGCGGTCGATCTTGCTGAA	CCGTTGCACGATTTCTACTGC
28	CGATGAGGCGGATCTGCTTG	CAGCGAGGTCTTGAAATACACAC
28	TCCTGTTGAAGTCGGCTTCAC	AAGAAGAAGAAGGTCATTGTGATCCC
28	CCAGGCAGCCTCTTGATCTC	GCGTTCGTCCTGGAAGGAT
28	CCAGGATTAGAACCCGAGTCGTA	GGTCTACATGGTCACCGGAAATC
27	TAGGACTTTTTACCCAAAACCTAGTCTCTA	CTCCCTTTTAGTTAAAAATGAGGCTTCC
26	GAAGCAAGACCTAGAAGCACCTT	TCTCTAATATTGGTGACGCCCATAGT
25	CTTGGCCATCCAGTCCCTAC	CCCAGACTAAGAGCCAGAGTTC
24	CACACTCTGTGTCCATACCACC	TAGAGCTTGCTTCCTGGAATGTC
23	GAAGCAAGCTCTAGGGCTCTG	GAATGGGTCTTGGCAATGGC
22	ACCTACGATCAGGGAGCAGAAA	GTGTAGAGTGGAGGGAGGATCT
21	ATCCTCTTTAATGGCTGTGCGTTA	GCAAGATCCTGTGACACGTACT
20	GGGAAGTACTCCAGTAGAAACCAGA	GAGGCACCCAGAGTATTCTGTG
19	GAAAGGCAGAGAGTAACCAGGTT	CGTAGGCTCAAGTCTCAGACAA
18	GTTTAGCCCTTGTTTCTTCCTCTCT	TCTGAGTCTCTTGAATTTAGTCACAGACT
17	CCTCAATGGTCGGGATTGACAC	AGATGTGGAGGCAGCGAGTATA
16	CCCAGCTCTGCTGTTTTAGAGG	AGTCCTTGCTGTCCAACATTCC
15	GCAGGAGCACACGTCGTA	GCAGCACTGGGCTATTTCCA
15	TAGGCCTCCAGGTAGGAGGAG	GCTACGACGTGTGCTCCTG
14	CACTAATGTGGAGACCTCGAGATT	CTAAACAACTATGCCGCTGCTTT
13	GGTGTAGGCCATGAGGAGAAAG	CCTAAGTCATTGCTCTTCAGTGCTA
12	GCATGCAGGTCCTTAAGGACAG	GAGAATGGAGTTGTAGGTTATGAGAAGG
11	TCCCTCATGCACAGAAAGCAAT	AAAGGACGTCCATGCAGTTTTG
10	GGCCTGTGAATGGGTTAGCATA	GATTCTGCTAGCACCAGCTCTT
9	CTCCAACTCAGTCTCTTCTTTCTTGG	GATAAACACCACAGAACAAGTTCTTTGAG
8	GCATCGCTCCTGACACATTTC	CAAGATGGAAGATGTTCATCTAAGGGA
8	CCACCCTCCAATTTCCCTCAAC	GTATGGAGTATAGGCAGTGTGTGT
7	TAAAGCCGCACATACGTGACA	ACTCAGGGAGACACTAACGGA
6	AATTAGACTCTAGCCACAGTTAGTTTTGG	AAAACCACCAGCAGACCTAGAATT
5	CCAGGGAAGGCATGTTAGTGAA	GGGTTAGAAGGTGGGAGAGACA
4	CCTGCTACTCACTTCCTTGGAA	GCTCGCTTGTAAAGACTTTTTGGG
3	GTTCCTCTCCTTGTTCTCAGCA	GCCAGAGAGGTTTGAGCTGATG
2	GCACCTGGTCTCTGGAATACAA	GGGTCACGAGGATCAATCTTTTCT
1	CTATCCCTACACCCCCACACT	CAGCAAAATTGGCTCAGGTTGA
1	AGATAAAGCCCAAGCTGTGACAT	CACTCTAGGAAGACCAGGGATCA
Promoter	CTCCATGATACCTCCTGCCCTA	GGCCTTTTCTACTGTCTCATGCT
Promoter	CTCAACACCATCTGCTAAACTAATTCCT	GGAACCTAGTGGTTTCTCAAAGTCT
Promoter	CAAGCCTAAGCCTTTGTAAAATGGT	TGCCCATTGCATCAGTTACTTATTTTAGT
Promoter	ACTCCAGGGAAGTTGAGAAAAACAC	AAACCATCACTCCCCACCTTC
