# Supplementary material for: Genetic Variation in the von Willebrand Factor Gene in Swedish von Willebrand Disease Patients
Source: TH Open. 2018 Jan 30;2(1):e39–48. doi: 10.1055/s-0037-1618571 (PMC6524857; doi:10.1055/s-0037-1618571)
Supplement: Supplementary file 2 — Supplementary Table S2 [file 10-1055-s-0037-1618571-s170015-2.pdf]

Table S2 Sanger sequencing primers for *VWF* and *ABO*

Exon	Primer sequence	
	Forward	Reverse
1	ATTCAGCTGCTCCCAGGTTC	GGTGTAGGGATAGGGGGTGT
2	CTTCCTCCTCATCCTGTGCC	CGATTGTTACCCTTGGGCCT
3	GCAACGGACACAGTGGACTA	CGAGGCCAGAGAGGTTTGAG
4	ACAAAGCCATAGGCCTCACC	TTTTGGGGCGTTTTCTGCTG
5	GCCAGGGAAGGCATGTTAGT	GGGGACCAAAGGTAAGCCAA
6	CTACACCAAGTCATTAGCCCA	GCAGACCTAGAATTTTCACCCA
7	TTTGGGAAGCCCGTTTGACA	CGGGTCCAGCATCTTTTCCT
8	CAACGGGGAGCAAACACAAG	GGACCTGGAAGCCTGAAGAC
9	TTTGCTCCCCATCATTGCCT	CCCCTACTGGATGACCTGGA
10	GTGCAGAGAGGGCAACTTCT	TGTTGGGGTTTCTTCCCCTG
11	ATGGGCTGGGTTTCTGGATG	GACATGGCAGTGAGCTGTGA
12	ACCTGCCCAAGACTCAACTC	TGCACTTAGCAAGCCTTCTT
13	GCCAATCTGGCTCTTCCCTT	GTGTCCCAGGCACATTCAGA
14	CTTTCCCGGGGGTTGGC	TTTGGGAACAGGGATGGAGC
15	GGAAACCCAAGCTTTGCAGG	GCAGCCCTCTATTAGCAGCA
16	CCTACTGCCAGCCAGGAATC	GTCACAGCTACAAGGGGTGG
17	CGTGAGGAATCTGGGCAGG	GTGGAGGCAGCGAGTATAG
18	CAGCACCACCTCCATTGCTA	AAGCCCAGGTGAGAAGATGC
19	GCAGGATGGACACAGGTGAT	AGACAACACTTCCTGGAGCG
20	TCTCCCACACCAATCCTCAAC	CTTCCTTGGGCTTCCTGGAG
21	GGTTCTTCCCACGTTTGTGC	CTGCAGGCACCTGGCTC
22	TCAGGGAGCAGAAAACACTCC	GAGTGGTGGTGGTGAGATGG
23	CTTCCAGCCCCCATGACAAT	TGCTTGTTGGGGACAGTGAT
24	CTAAGACTGGGGCTGAAAGAGAAG	GGTTGTGGGTGGTATGACCTC
25	TTGGCCATCCAGTCCCTACT	TTGCCTTCCCACTCATTCCC
26	CATAGGATCGTCACAAACTGCG	TTCTTTGGACAGAAGCAACTTAATGT
27	GACTTTTTACCCAAAACCTAGTCTCTA	CCTTTTAGTTAAAAATGAGGCTTCCTC
28,1	TTGGGCCCCAGGGTCGAAAC	GGGCTCAGAAGTGTCCACAGGTTC
28,2	CCAGGATTAGAACCCGAGTCG	AGGCCTTCGTGCTGAGCA
29	TCCATACTGCAGCACTGACA	ACATTGCCCTTGTACTCACGA
30	GGCTCGACACCCTGTCTT	TGTAGGCCTGGTGGCCAT
31	CATCCAAAAGTAACCCCAGC	ACCGTTAAGACAGGGTGTCG
32	CTCTGCCAAGTTCACATGGA	TCAGGCCAGTCCATTTTGAG
33	ACAAGATGTACAGATGGACCCG	ACCTCAGCCTCATGTCCCTAT
34	CTCAGAAAAGCAATTCTTCCTTCCA	CTCCTTGCTGTGTAGGCCT
35	TCCCAATTCTCCACCAGTGC	TGTGCAACTTGGTCACACCT
36	GATAAACTGAGTGGCCCTGG	TTGCCCTTCCCTGTTGAGTC
37	AGCTGGTCTCCAGGATTTTCA	AAGAATGGCCTGTCTCTGGC
38	CCACAGTTGGAAGAGGCCAA	CCATGTTGAATCAGCTGTGCC
39	AGGCCCAGTGATGGGAAATG	TCAGCCTGCTTTTGTTTGCC
40	ACCCACCTCCTTTCACACAC	CATTTCCCATCACTGGGCCT
41	TAAACCCAAGACGGAGGTGC	TGTAGCATCCCACTCACAGG
42	TGTAGCACTTGGTTTGGGCA	GGGATTGAGCAGCCTTCACT
43	CACCCCTAGCCTTGAACCAG	GGAAGAAGGGAGACCAAGGC
44	CAACAGCTGGGTGAAATGCC	GCTAAGGGAACTCTGGGCTG
45	AGCAGTAGGAGCAGATGGGA	CAATGCTGAGGAAGGGAGGG
46	GGCCAGGAAGTGAGTGTGAG	GGCTCTTCTCATTGTGGGCT
47	ATGCAGTTTGGGTGGGTGAT	AGGGTATGAGAGTGAGGTGAG
48	TTGTGAGGCCCTACACCAAG	AGAGAGATGAGAGGCCAGCA
49	CAGCTTGACCACCCTACCTG	CTGGGTGTGGGGGCTTTATT
50	GTCATGCGGCTTGCTAATGG	CAGGTAGGGTGGTCAAGCTG
51	TTTCCCTTCTCTTCCCTGCG	ACAAGCACAAGAGGGTTGCT
52	AAGAGGCCTACCCCATGCTA	CTAGCTGTCCTGGGTGCTTC
ABO exon 7	GTATTGAGGGGGTGGCTCAG	AGGTACCTCCTACCTGGGGA
