# Supplementary material for: Genetic Variation in the von Willebrand Factor Gene in Swedish von Willebrand Disease Patients
Source: TH Open. 2018 Jan 30;2(1):e39–48. doi: 10.1055/s-0037-1618571 (PMC6524857; doi:10.1055/s-0037-1618571)
Supplement: Supplementary file 3 — Supplementary Table S3 [file 10-1055-s-0037-1618571-s170015-3.pdf]

Table S3 Sequenom genotyping of 98 SNPs in 106 index cases ($VWD1_{106}$) and allele frequency comparison with CEU population

		MAF		*P*-value‡
SNP ID	Chr. position*	1000G†	$VWD1_{106}$	$VWD1_{106}$ / 1000G
rs2362481	6049161	50.0	43.4	0.99
rs12580835	6049784	47.0	41.0	0.81
rs7310736	6052265	43.4	40.6	0.89
rs7314566	6052868	35.9	44.4	0.68
rs11063951	6056668	18.7	24.3	0.35
rs1990326	6057911	43.4	40.1	0.38
rs933408	6058736	21.2	14.8	0.29
rs2286646	6061491	22.2	24.1	0.16
rs7962217	6061559	6.1	3.3	0.44
rs7969672	6063165	21.7	14.6	0.26
rs11063953	6065070	18.2	23.8	0.26
rs10849362	6066261	33.8	35.1	0.64
rs10849363	6066357	39.9	38.6	0.84
rs2363309	6070839	39.4	37.5	0.75
rs12369177	6073520	33.3	36.7	1.00
rs11063961	6074016	24.7	25.2	0.13
rs4764478	6078125	24.7	22.2	0.32
rs35335161	6078424	4.5	3.8	0.66
rs2270239	6078616	30.8	34.4	0.77
rs11063965	6080595	28.3	34.0	0.67
rs917857	6081824	49.5	43.4	0.59
rs917858	6081938	29.3	34.8	0.83
rs4764521	6084323	21.7	16.5	0.49
rs216855	6085513	24.7	30.2	0.14
rs216856	6086265	32.8	35.8	0.43
rs216865	6090303	32.8	36.2	0.48
rs216867	6091000	13.1	15.1	0.35
rs2058473	6093926	37.4	35.8	0.51
rs216883	6097657	26.3	19.6	0.17
rs216893	6100485	49.5	49.1	0.75
rs216896	6100796	49.5	49.1	0.75
rs12579603	6102125	37.4	36.2	0.46
rs34230288	6103094	3.5	1.0	0.84
rs216902	6105387	37.9	33.5	0.36
rs216903	6105499	49.5	50.0	0.94
rs216904	6106018	37.9	33.5	0.36

rs216905	6106255	23.7	20.3	0.44
rs216801	6108513	37.9	33.3	0.37
rs216809	6114283	38.4	33.2	0.29
rs216811	6115274	32.8	25.7	0.32
rs216812	6117211	32.8	26.4	0.23
rs542993	6120431	49.0	41.0	0.09
rs1800385	6127891	8.1	9.0	0.61
rs216312	6128984	46.0	38.6	0.62
rs56068059	6134987	23.2	18.6	0.54
rs11611917	6136634	23.2	18.6	0.54
rs11612384	6136846	23.2	19.5	0.37
rs1800380	6138595	23.2	19.3	0.40
rs60831677	6138702	9.1	7.6	0.55
rs33978901	6140659	3.5	0.9	0.83
rs216322	6144897	33.3	29.5	0.63
rs216326	6145948	42.4	37.1	0.90
rs216327	6146149	33.3	30.5	0.46
rs216332	6147056	33.3	29.5	0.63
rs1860365	6147110	23.2	19.0	0.26
rs216336	6148230	35.9	31.9	0.42
rs10849378	6148674	24.7	20.8	0.21
rs216339	6149284	10.6	11.9	0.90
rs2239162	6151670	30.3	26.9	0.19
rs1063857	6153514	35.4	30.0	0.22
rs1063856	6153534	35.4	30.0	0.22
rs216293	6153659	47.0	42.4	0.38
rs7954351	6163705	30.3	24.3	0.49
rs980131	6169198	38.9	43.8	0.66
rs4764482	6169733	48.0	49.5	0.89
rs1800378	6172202	39.4	34.5	0.43
rs1800376	6174414	11.6	13.7	0.35
rs1800375	6174423	11.6	13.7	0.35
rs2239153	6186667	43.9	48.6	0.60
rs2238104	6187665	42.4	45.8	0.15
rs763580	6193537	35.9	30.5	0.90
rs2238103	6194183	39.4	37.6	0.24
rs2238101	6198231	35.9	31.6	0.89
rs1034933	6198940	34.8	31.6	0.67
rs2239140	6200443	49.0	40.1	0.59
rs11064024	6202049	40.9	40.0	0.25
rs12317079	6202462	40.4	42.4	0.38
rs7306706	6215634	38.9	48.1	0.85
rs3782716	6222087	32.8	39.6	0.70

rs12306928	6226301	34.3	40.5	0.19
rs2286608	6232426	34.3	40.3	0.12
rs10849387	6238479	32.8	36.2	0.12
rs10774394	6238593	33.3	38.5	0.14
rs6489695	6255425	29.8	32.1	0.03
rs7979747	6268288	34.8	36.2	0.12
rs10774401	6272849	35.9	37.9	0.10
rs10849406	6275669	35.9	36.8	0.09
rs7956209	6278660	35.4	36.7	0.15
rs10744699	6280584	33.8	32.4	0.25
rs11064074	6281039	46.0	49.0	0.84
rs10774407	6286080	47.5	49.5	0.84
rs1558327	6289067	41.4	43.4	0.77
rs1558325	6289108	41.9	43.4	0.77
rs887475	6292691	46.5	44.2	0.96
rs2363880	6302009	46.0	42.9	0.92
rs797773	6307566	42.4	38.7	0.17
rs797775	6311160	46.5	39.5	0.31
rs758730	6314243	45.5	50.0	0.87

*According to GRCh37p13.

† The CEU population (99 individuals) of the 1000Genomes project.

‡ After Bonferroni correction $\alpha_{corrected}$ = 0.05/98 = 0.00051
